# The expression of tumor necrosis factor-alpha, its receptors and steroidogenic acute regulatory protein during corpus luteum regression

**DOI:** 10.1186/1477-7827-6-50

**Published:** 2008-11-07

**Authors:** Michael Abdo, Susan Hisheh, Frank Arfuso, Arun Dharmarajan

**Affiliations:** 1 School of Anatomy and Human Biology, The University of Western Australia, 35 Stirling Highway, Crawley, Western Australia 6009, Australia

## Abstract

**Background:**

Corpus luteum (CL) regression is known to occur as two parts; functional regression when steroidogenesis declines and structural regression when apoptosis is induced. Previous studies suggest this process occurs by the production of luteolytic factors, such as tumour necrosis factor-alpha (TNF-alpha).

**Methods:**

We examined TNF-alpha, TNF-alpha receptors (TNFR1 and 2) and steroidogenic acute regulatory (StAR) protein expression during CL regression in albino Wistar rats. CL from Days 16 and 22 of pregnancy and Day 3 post-partum were examined, in addition CL from Day 16 of pregnancy were cultured in vitro to induce apoptosis. mRNA was quantitated by kinetic RT-PCR and protein expression examined by immunohistochemistry and Western blot analyses.

**Results:**

TNF-alpha mRNA increased on Day 3 post-partum. TNFR were immunolocalized to luteal cells, and an increase in TNFR2 mRNA observed on Day 3 post-partum whilst no change was detected in TNFR1 mRNA relative to Day 16. StAR protein decreased on Day 3 post-partum and following trophic withdrawal but no change was observed following exogenous TNF-alpha treatment. StAR mRNA decreased on Day 3 post-partum; however, it increased following trophic withdrawal and TNF-alpha treatment in vitro.

**Conclusion:**

These results demonstrate the existence of TNFR1 and TNFR2 in rat CL and suggest the involvement of TNF-alpha in rat CL regression following parturition. Furthermore, decreased StAR expression over the same time points was consistent with the functional regression of the CL.

## Background

The demise of the corpus luteum (CL) is characterized by a decrease in progesterone synthesis [1] and an increase in apoptotic cell death [2]. Whilst a temporal pattern is well established, the factors regulating both the functional and structural regression of the rat CL remain poorly understood.

Whilst progesterone is synthesized by the ovary, the adrenal and the placenta, the CL of pregnancy are the major source of progesterone in the rat [3-5]. Small and large luteal cells within the rat CL of pregnancy retain steroidogenic potential though large luteal cells predominate [6]. During pregnancy total progestin synthesis (progesterone and 20α-hydroxypregn-4-en-3-one (20α-OHP)) declines from a high on Day 16 to the morning of Day 22 prior to an increase in the afternoon on Day 22 [1]. This observed pattern in total progestin production in rats has been demonstrated to be a product of decreased synthesis of progesterone toward term [7] and increased synthesis of 20α-OHP [1].

Total progestin production is dependent on the transport of cholesterol to the mitochondria and then from the outer to the inner mitochondrial membrane which is mediated by the steroidogenic acute regulatory (StAR) protein [8]. StAR protein has been reported in the ovary of the mouse [9], rat [10], rabbit [11] and human [12] and correlated with the functional state of the CL [11,13,14]. As such StAR expression has been proposed as a reliable "marker" of CL function [15].

Several publications have reported the participation of the immune system in ovarian events [16], suggesting a role for the cytokine tumor necrosis factor – alpha (TNFα) in CL regression. Luteal cells and endothelial cells are capable of TNFα synthesis though macrophages remain the primary ovarian source [17,18]. TNFα expression in the ovary is coordinated between the infiltration and activation of macrophages and the hormonal regulation of the CL [19-21]. We have recently reported TNFα protein localization in the rat CL on Day 16 and Day 22 of pregnancy and Day 3 post-partum [22]. Furthermore, we have demonstrated the induction of luteal cell apoptosis following treatment with recombinant TNFα in a dose- and time-dependent manner [22].

Associated with the TNFα ligand are two similar, though distinct receptors, TNFα receptor 1 (TNFR1) and TNFα receptor 2 (TNFR2). The lack of homology between the two cytoplasmic domains [23,24] is thought to contribute to the different outcomes of TNFα. Involved in a variety of biological processes, TNFα is implicated in both cell proliferation and cell death; TNFR1 is generally associated with TNFα-induced cell death and TNFR2 with cell proliferation [25]. TNFR binding sites have been demonstrated within the bovine [18,26], porcine [27] and rat [28,29] CL under various experimental conditions. TNFR are present on nearly all cell types with few exceptions [24] and the subtypes are often co-expressed by the same cells [30].

The aims of this study are to examine the role of TNFα during the structural regression of the CL by analysis of TNFR expression, and to determine the role of TNFα in the functional regression of the CL through regulation of StAR protein expression.

## Methods

### Animals

The animals used were mature (12–20 week old) nulliparous albino Wistar rats obtained from the Animal Resources Center (Murdoch, WA, Australia). Animals were housed in a controlled environment and mated overnight. Day 1 of pregnancy was designated as the morning on which spermatozoa were present in a vaginal smear. Litters were born on Day 23 of pregnancy. All procedures were approved by The University of Western Australia Animal Ethics Committee.

### Experimental tissue collection

All tissues were collected under aseptic conditions with light anesthesia using a mix of 0.2 L/min O_2_, 0.8 L/min NO and 5% Halothane. CL were collected on Day 16 and Day 22 of pregnancy and Day 3 post-partum. Four rats from each stage of pregnancy and post-partum were used. One ovary from each animal (alternating left or right) was used for immunohistochemistry, the contralateral ovary was used for Western blot and mRNA analyses (n = 4). Ovaries were trimmed of adhering tissues and the CL of pregnancy were selected and dissected as previously described [2]. In addition to the above, *in vitro *studies were conducted using CL collected on Day 16 of pregnancy. Three pairs each from a different animal (n = 3) were collected for each treatment group. Dissected CL were cultured in MEM either without trophic support for 8 h or with minimal trophic support supplemented with 37.5 ng/ml of recombinant rat TNFα (R&D Systems, USA) for 6 h as described previously [22]. Following incubation protein or mRNA was extracted from each CL pair as described under each experimental method.

### Immunohistochemistry

CL collected for immunohistochemistry were fixed and processed as previously described [22,31]. Sections were treated for 10 min with 3% hydrogen peroxide in methanol, washed in PBS (pH 7.4) and treated with 10% fetal bovine serum (FBS; Sigma Chemical Co., St Louis, MO, USA) (TNFR) or 0.1% bovine serum albumin (BSA; Sigma Chemical Co.) (StAR) for 1 h at room temperature. Sections were incubated with either 1:50 polyclonal goat anti-rat TNFR1 antibody (Santa Cruz Biotechnology, Santa Cruz, CA, USA), 1:100 polyclonal goat anti-rat TNFR2 (Santa Cruz Biotechnology) or 1:100 polyclonal rabbit anti-mouse StAR antibody (supplied by Professor Doug Stocco). TNFR antibodies were diluted in PBS pH 7.4 whilst the StAR antibody was diluted in PBS pH 7.4, 1% BSA, 0.1% Triton X. Sections were incubated for 2 h at 37°C (TNFR) or overnight at 4°C (StAR). Following this, sections were incubated for 1 h at 37°C with a 1:200 donkey anti-goat HRP (Santa Cruz Biotechnology) secondary antibody (TNFR) or 1:200 biotinylated goat anti-rabbit IgG (Santa Cruz Biotechnology) secondary antibody (StAR). The sections were then incubated with Avidin Biotin Enzyme Reagent (Vector Laboratories, Burlingame, CA, USA) for 1 h at room temperature (StAR) and the reaction visualized by the addition of 3,3'-diaminobenzidine tetrahydrochloride (DAB; 1.2 mg/ml). The immunohistochemical procedures described were repeated for each animal group (n = 4).

### Western blot analysis

CL collected for Western blot analyses were snap frozen in liquid nitrogen and stored at -70°C until use. Total protein was extracted by homogenization in RIPA buffer (150 mM NaCl, 50 mM Tris-HCl, pH7.5, 1% Triton X, 0.5% Na deoxycholate, 1 mM PMSF) as described previously [32]. Protein concentration of homogenates was measured [33] and 30 μg resolved by 12% SDS-PAGE and transferred to nitrocellulose membranes (MSI, Westboro, MA, USA).

Membranes were blocked in 5% non-fat milk for 1 h at room temperature and probed with polyclonal rabbit anti-mouse StAR antibody diluted 1:5000 in Tris-buffered saline/0.1% Tween-20 (TBST). Following this membranes were incubated with biotinylated goat anti-rabbit IgG for 1 h at room temperature (diluted 1:10,000 in TBST). Membranes were then incubated with Avidin Biotin Enzyme Reagent for 1 h at room temperature and protein signals detected by enhanced chemiluminescence (Supersignal West Pico ECL substrate, Pierce, Rockford, IL, USA) and quantitated by densitometry.

A common tissue sample was included on each gel to allow for standardization of chemiluminescence levels and exposure times. Staining of each gel (post transfer) and membrane with Coomassie Brilliant Blue (Sigma Chemical Co.) assessed the accuracy of sample loading and the efficiency of protein transfer. This procedure was repeated for each animal/experimental group (n = 3).

### Kinetic RT-PCR

TNFα, TNFR1, TNFR2, L19 and StAR mRNA expression were quantitated through kinetic reverse transcription (RT) – polymerase chain reaction (PCR) using the Bio Rad iCycler (Bio Rad Laboratories, Hercules, CA, USA). All tissue collected was snap frozen in liquid nitrogen and stored at -70°C until use. Total RNA was extracted using RNAzol B (Tel-test, Friendswood, TX, USA) and 5 μg reverse transcribed using SuperScript II reverse transcriptase (Invitrogen, Life Technologies, Melbourne, Australia) as per manufacturer's instructions. cDNA samples were purified using an UltraClean PCR kit (Mo Bio Laboratories, Solana Beach CA, USA), concentrations measured by spectrophotometry and samples stored at -20°C until use.

Kinetic PCR and melt curve analyses were performed using the Qiagen Quantitect PCR SYBR Green I kit (Qiagen, Clifton Hill, Victoria, Australia) according to manufacturer's instructions with the addition of 100 nM fluorescein (Bio Rad Laboratories). 2.5 μl of each RT sample (cDNA) was added to the 1× PCR master mix in a 25 μl final volume. Primers used for each target (0.5 μM) were based on published rat sequences (Table 1). Each PCR cycle included an initial denaturation at 95°C for 15 min (including 90 sec at 95°C for automated well factor collection) followed by 45 cycles of 95°C for 30 sec, 52–57°C (depending on target) for 30 sec, and 72°C for 60 sec. The annealing temperatures used for each target were; TNFα 56°C, TNFR1 52°C, TNFR2 54°C, L19 56°C and StAR 52°C. A fluorescence measurement was performed during the extension step of each cycle. In addition, melt curve analysis was performed with continuous fluorescence measurement between 55–95°C in 0.5°C increments.

**Table 1:** Primer sequences used for individual targets

**Target**	**Forward Primer**	**Reverse Primer**
TNFα Clontech Laboratories Inc, CA, USA	5'-TAC TGC ACT TCG GGG TGA TTG GTC C-3'	5'-CAG CCT TGT CCC TTG AAG AGA ACC-3'
		
TNFR1 [28]	5'-CCA GCC CCA ATG GGG GAG TG-3'	5'-CGG TGT TCT GTT TCT CCT TA-3'
		
TNFR2 [28]	5'-TTC GGA GTG GCC CGT TCA AGA-3'	5'-GCT GTG GTC AAT AGG TGC TGC-3'
		
L19 [36]	5'-CTGAAGGTCAAAGGGAATGTG-3'	5'-GGACAGAGTCTTGATGATCTC-3'
		
StAR [32]	5'-GCA GCA GGC AAC CTG GTG-3'	5'-TGA TTG TCT TCG GCA GCC-3'

External standards for each target were generated by extraction of the RT-PCR product following agarose gel electrophoresis using the QIAquick PCR Purification Kit (Qiagen) as per manufacturer's instructions. Samples were quantified by spectrophotometry, and then used to generate a standard curve via serial dilutions. Fluorescence data were analyzed and a standard curve generated using the Bio Rad iCycler software (3.0 beta) (Bio Rad Laboratories).

The potential for genomic DNA contamination was assessed by amplification of a DNA sample and RT controls (no RNA template). To confirm reproducibility, repeats (n = 3) for each time-point of interest were amplified in duplicate, the external standards were amplified in duplicate simultaneously. To avoid competition, target and L19 cDNAs were amplified in 2 separate PCR reactions. At the completion of each PCR reaction the starting quantity of each sample was calculated against the standard curve (constructed by software) using the appropriate threshold cycle (C^T^) value. Samples were given a relative measure against their starting cDNA concentration [34] and this value normalized against corresponding L19 value [35,36].

### Statistical analyses

All experiments were conducted using a minimum of three animals per time point/treatment. Variation among groups was analyzed by one-way ANOVA or t-test where appropriate. Where significant differences (*P *< 0.05) among groups were detected, specific group comparisons were made by least significant difference (LSD) tests [37]. Associations between parameters were measured by Pearson correlation.

## Results

### Immunohistochemistry

**Figure 1:**
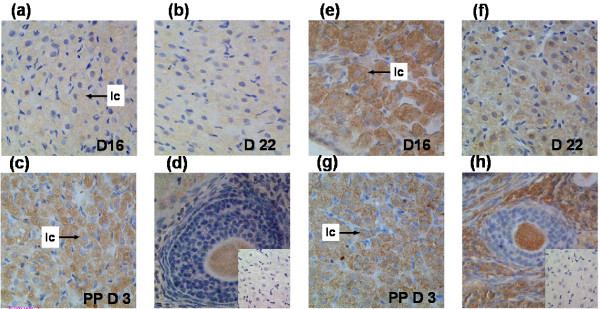
**TNF receptor 1 & 2 immunohistochemistry**. Rat CL sections incubated with polyclonal goat anti-rat TNFR1 or TNFR2 antibody stained with DAB and counterstained with haematoxylin. TNFR1 protein expression was assessed on Days 16 (a) and 22 (b) of pregnancy and Day 3 (c) post-partum. Oocytes at all stages of follicular development were immunoreactive (d) The staining pattern and intensity were consistent between immunohistochemical runs (n = 4) and staining intensity was greatest in Day 3 post-partum sections (c). TNFR2 protein was also assessed on Days 16 (e) and 22 (f) of pregnancy and Day 3 post-partum (g). Immunoreactive-TNFR was observed across all time-points within the CL compartment and specifically luteal cells (lc). Oocytes at all stages of follicular development were immunoreactive (h). Sections treated in the absence of the primary antibody showed no non-specific immunostaining for TNFR1 (inset d) or TNFR2 (inset h). [Magnification 400×]

Immunohistochemical staining indicated that TNFR1 and TNFR2 (Figure 1) were present in the rat CL. Immunoreactive-TNFR1 and TNFR2 were evident in CL on Days 16 and Day 22 of pregnancy and post-partum Day 3. The staining intensity of TNFR1 appeared to increase on Day 3 post-partum whilst the staining intensity of TNFR2 was highest on Day 16, then reduced on Day 3 post-partum and least intense on Day 22 of pregnancy. Within the CL, immunostaining was concentrated in the cytoplasm of luteal cells. Cells within the ovarian interstitium and oocytes at all stages of follicular development were immunoreactive for TNFR1 and TNFR2. Sections treated in the absence of the primary antibody showed no immunostaining.

**Figure 2:**
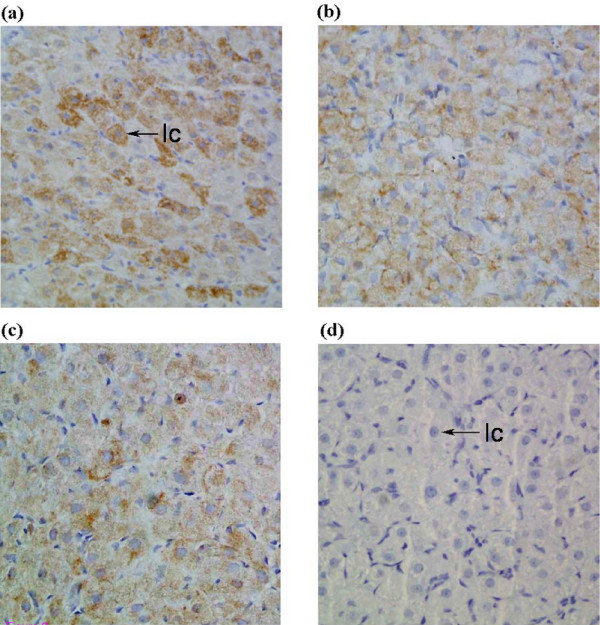
**StAR protein localization**. StAR protein expression was analyzed using a polyclonal rabbit anti-mouse StAR antibody on Day 16 (a) and 22 (b) of pregnancy and Day 3 post-partum (c). Immunoreactive-StAR was observed across all days in the CL compartment, specifically in luteal cells (lc). Sections treated without the primary antibody showed no immunostaining (d). The staining pattern and intensity were consistent between immunohistochemical runs (n = 4). [Magnification 400×]

Immunoreactive-StAR was evident in CL at all stages of pregnancy and post-partum examined (Figure 2). StAR protein was localized within the cytoplasm of luteal cells on Days 16 and Day 22 of pregnancy and post-partum Day 3. Staining intensity appeared to decrease toward Day 3 post-partum. Negative control sections incubated in the absence of the primary antibody showed no immunostaining. The staining pattern and intensity for all protein targets were consistent between immunohistochemical runs (n = 4).

### Western blot analysis

**Figure 3:**
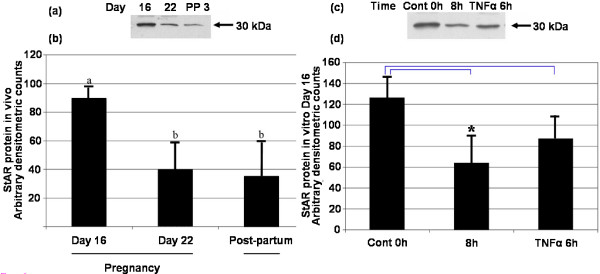
**Western blot analysis of StAR protein in vivo and in vitro**. Western blot analysis of StAR protein on Day 16 and 22 of pregnancy, and Day 3 post-partum. A representative autoradiogram showing a single immunoreactive band (a) and corresponding statistical analysis (b). There was a significant change between Day 16 of pregnancy and Day 3 post-partum (*P *< 0.05; one-way ANOVA). Values without shared notations differ at *P *< 0.05 (LSD test). Western blot analysis of StAR protein on Day 16 of pregnancy following incubation without trophic support for 8 h or following treatment with recombinant rat TNFα (37.5 ng/ml). A representative autoradiogram showing a single immunoreactive band (c) and corresponding statistical analysis (d). Values are expressed in arbitrary density units and show mean ± SEM for all groups (n = 3). Asterisk denotes a significant difference between control and 8 h group (*P *< 0.05; t-test).

Western blot analysis revealed a single immunoreactive band of approximately 30 kDa consistent with that previously reported [38] (Figure 3a). StAR protein expression decreased significantly (*P *< 0.05) between Day 16 and the other time points examined. StAR protein expression decreased significantly following *in vitro *incubation without trophic support for 8 h (Figure 3b). There was no significant change in StAR protein expression following treatment with recombinant TNFα (37.5 ng/ml) for 6 h (Figure 3b).

### Kinetic RT-PCR

**Figure 4:**
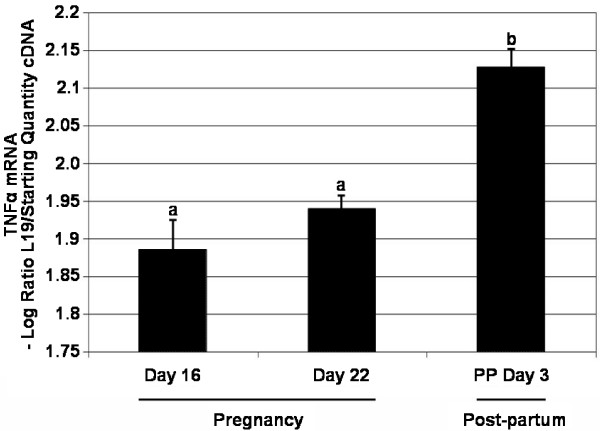
**TNFα mRNA expression**. TNFα mRNA expression was assessed through kinetic RT-PCR on Day 16 and 22 of pregnancy and Day 3 post-partum. mRNA levels were normalized against L19 and are shown as mean ± SEM for all groups (n = 3). There was a significant difference between pregnancy and post-partum time-points (*P *< 0.05; one-way ANOVA). Values without shared notations differ at *P *< 0.001 (LSD test).

TNFα mRNA RT-PCR product was detected in ovarian samples on all days/time-points (Figure 4). Approximately 25–30 amplification cycles were needed to reach the threshold, the threshold cycle (C^T^) vs. log (starting concentration) plot or standard curve was linear with a strong correlation coefficient (*r *= 0.999) (data not shown). The relative amount of TNFα increased significantly on Day 3 post-partum (*P *< 0.001) compared to Day 16 of pregnancy, though there was no significant difference between Day 16 and Day 22 of pregnancy (Figure 4). Melt curve analysis revealed the amplification of a single product (295 bp) with a denaturation temperature (Tm) of 87°C (data not shown).

**Figure 5:**
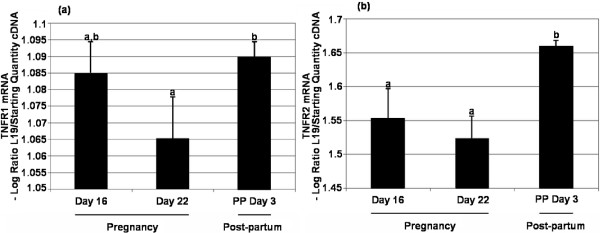
**TNFR1and TNFR2 mRNA analysis**. (a) Kinetic RT-PCR measurements of TNFR1 mRNA expression on Day 16 and 22 of pregnancy and Day 3 post-partum. There was a significant difference between Day 22 of pregnancy and Day 3 post-partum groups (*P *< 0.05; one-way ANOVA). Values without shared notations differ at *P *< 0.05 (LSD test). (b) TNFR2 mRNA expression was assessed on Day 16 and 22 of pregnancy and Day 3 post-partum by kinetic RT-PCR. mRNA levels were normalized against L19 and shown as mean ± SEM for all groups (n = 3). There was significant variation between pregnancy and post-partum (*P *< 0.05; one-way ANOVA). Values without shared notations differ at *P *< 0.05 (LSD test).

A single RT-PCR product corresponding to both TNFR1 and TNFR2 on Day 16 and Day 22 of pregnancy and Day 3 post-partum. Approximately 21–27 (TNFR1) and 23–26 (TNFR2) amplification cycles were needed to reach the threshold and the standard curves generated were linear with strong correlation coefficients (*r *= 0.999 and 0.989 respectively) (data not shown). There was a slight but insignificant change in TNFR1 mRNA levels between Day 16 and Day 22 of pregnancy (Figure 5a), but levels increased significantly (*P *< 0.05) from Day 22 of pregnancy to Day 3 post-partum. TNFR2 mRNA levels increased significantly (*P *< 0.05) on Day 3 post-partum compared to Day 16 and Day 22 of pregnancy (Figure 5b). There was no significant difference in TNFR2 mRNA levels between Day 16 and Day 22 of pregnancy. Following amplification, samples were subjected to melt curve analyses which demonstrated a single product of 536 bp with a Tm of 88°C (TNFR1) and 527 bp with a Tm 86°C (TNFR2) (data not shown).

**Figure 6:**
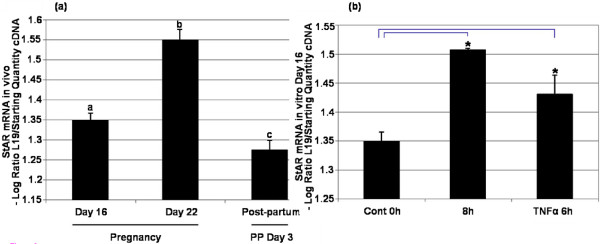
**StAR mRNA analysis in vivo and in vitro**. StAR mRNA expression measured by kinetic RT-PCR on Day 16 and 22 of pregnancy and Day 3 post-partum. mRNA levels were normalized against L19 and are shown as the mean ± SEM for all groups (n = 3). There was a significant difference among groups (*P *< 0.05; one-way ANOVA). Values without shared notations differ at *P *< 0.05 (LSD test). (b) mRNA expression measured by kinetic RT-PCR on Day 16 of pregnancy following incubation without trophic support for 8 h or following treatment with recombinant rat TNFα (37.5 ng/ml). mRNA levels were normalized against L19 and are shown as mean ± SEM for all groups (n = 3). Asterisk denotes significant difference between control and treatment groups (*P *< 0.05; t-test).

StAR mRNA expression was assessed by kinetic RT-PCR on the same days of pregnancy and post-partum (Figure 6a). The amplification of StAR required approximately 20–25 cycles to reach the threshold and the standard curve generated was linear with a strong correlation coefficient (*r *= 0.985) (data not shown). The relative amount of StAR mRNA increased significantly from Day 16 to Day 22 of pregnancy then decreased to levels below Day 16 on Day 3 post-partum (*P *< 0.05) (Figure 6a). StAR mRNA expression was further examined following *in vitro *incubation without trophic support for 8 h and treatment with recombinant TNFα (37.5 ng/ml) for 6 h (Figure 6b). The relative amount of StAR mRNA increased significantly following incubation without trophic support and following treatment with recombinant TNFα (*P *< 0.05). Melt curve analysis revealed the amplification of a single product of 246 bp, with a Tm of 87°C (data not shown).

## Discussion

Our work to date has focused on elucidating the mechanisms of CL regression, particularly those associated with structural regression of the CL [22,31,39,40]. We have previously demonstrated TNFα expression during the structural regression of the CL both *in vivo *and *in vitro *through immunohistochemistry and Western blot analyses [22], concluding that TNFα is a potential luteolytic factor at Day 22 of pregnancy and Day 3 post-partum. The analysis of TNFα mRNA expression in this study supports these earlier findings and suggests that the involvement of TNFα in CL regression is active (requiring transcription) rather than passive.

Critical to the effectiveness of cytokine-mediated apoptosis, is receptor-ligand binding. The immunohistochemical data in this study demonstrate for the first time the presence of the TNFR in adult rat CL during pregnancy and post-partum. Since the TNFR is an essential element in the TNFα cell death pathway, our findings further support a role for TNFα during CL regression. TNFR immunostaining appeared to be confined to rat luteal cells, despite published evidence of endothelial cell expression within the porcine and bovine CL [18,27,41]. Furthermore, immunostaining was not solely localized to the cell membrane (as expected) but also the cytoplasm of luteal cells; for this reason quantitative analysis of protein expression was not undertaken since the results, whilst supporting the presence of TNFR in the CL, do not definitively define it's cellular compartmentalization. Importantly, this finding is supported by the manufacturer's (Santa Cruz Biotechnology) disclosure stating the presence of both membrane and cytoplasmic staining for either antibody. Indeed photographs presented in cited publications [42,43] clearly show both cytoplasmic and membrane-bound localization of TNFR1 and TNFR2. Whilst the significance of these findings is not discussed it is possible that it reflects either latent TNFR protein expression or is the result of the homology between the TNFR death domain and the death domain of adapter proteins localized within the cytoplasm.

TNFR mRNA was expressed in the rat CL at all stages examined during pregnancy and post-partum. One of the unanswered questions in TNFα biology is what types of signals are mediated through either TNFR. The hypothesis of this study was that CL fate might be regulated at the level of the TNFR; should only one receptor type be expressed during CL regression. There was no change in TNFR1 mRNA levels when compared to Day 16 of pregnancy; however, there was a significant increase in TNFR1 mRNA levels from Day 22 of pregnancy to Day 3 post-partum. This finding supports the association of TNFR1 with apoptosis [25] and also its association with the rapid luteal regression seen following parturition. TNFR2 mRNA was more abundant on Day 3 post-partum, a period when active growth of new follicles is occurring, and this finding further supports the cell proliferative role of TNFR2 [25]. Whilst mRNA expression cannot be extrapolated to protein expression, the observed changes are intriguing.

Despite its association with cell survival, TNFR2 expression may also contribute to the apoptotic signal of TNFα. Anti-TNFR2 antibodies, although not directly cytotoxic, can antagonize the cytotoxic action of TNFα [44]. Tartaglia et al., [45] hypothesized that the auxiliary function of TNFR2 was to cooperate in the binding of TNFα to TNFR1 (the ligand-passing hypothesis). An alternate hypothesis is that TNFR2 may be responsible for the DNA fragmentation activity associated with TNFα-induced apoptosis [46]. Although separation between the functioning of the two receptors has been demonstrated, overexpression of TNFR2 can result in apoptosis [47]. Thus the increased levels of TNFR2 mRNA observed on Day 3 post-partum may possibly contribute to TNFα-induced apoptosis [48]. However, if none of these hypotheses hold true, these data do not diminish the role of TNFα during CL apoptosis since, associated with receptor expression, is a reported increase in the binding affinity of TNFα during the oestrus cycle and pregnancy [26,28,49]. A quantitative study of TNFR binding affinity and protein expression in the rat CL is required before such conclusions can be made.

The role of TNFα in the functional regression of the CL was assessed through analysis of StAR protein expression. Localized to luteal cells, StAR protein and mRNA expression decreased on Day 3 post-partum. StAR expression correlated with the reported changes in total progestin synthesis [1] and the structural regression of the CL [40,50,51]. Whilst StAR expression was significantly reduced on Day 3 post-partum, the observed increase in mRNA expression on Day 22 of pregnancy is consistent with the observed synthesis of 20α-OHP [1]. Importantly, the decline in StAR expression post-partum was inversely correlated with TNFα expression as reported in earlier studies [15]. However, following treatment with recombinant TNFα, StAR mRNA expression increased whilst protein expression remained unchanged. A similar effect was observed following the withdrawal of trophic support for 8 hours. We have previously demonstrated the pattern of apoptosis occurring both during pregnancy and following *in vitro *organ culture [2,22] and the decline in progesterone synthesis during the structural regression of the CL is well documented [3]. It is possible that the elevation in StAR mRNA corresponds to the attempted compensation by remaining healthy luteal cells in a similar manner to that observed following unilaterally ovariectomized rats [52]. As such the *in vitro *data present confounding evidence for the role of TNFα in the functional regression of the CL.

## Conclusion

The results of the present study indicate the local production of TNFα and the presence of both TNFR in rat CL throughout pregnancy, and further support the role of TNFα in the structural regression of the rat CL. The data further demonstrate the relationship between StAR expression and the functional state of the rat CL and suggest that TNFα is associated with its functional regression. This work forms the basis from which further investigations around TNFα systems' biology may be undertaken and may ultimately lead to the ability to manipulate CL regression.

## Competing interests

The authors declare that they have no competing interests.

## Authors' contributions

This is part of MA's Ph.D work. MA carried out most of the experiments with the help of SH. FA participated in revising the manuscript for important intellectual content. AD was involved in the design of the experiments. All authors contributed to drafting the manuscripts. All authors read and approved the final manuscript.
